# Department of Surgery

**Published:** 1900-10

**Authors:** W. E. A. Wyman, J. A. Sloan, Robert Jay

**Affiliations:** Burlington, Wis.; San Francisco, Cal.; West Liberty, Ia.


### DEPARTMENT OF SURGERY.

By W. E. A. Wyman, M.D.V., V.S.,
BURLINGTON, WIS.

J. A. Sloan, B.Sc., M.D.V., SAN FRANCISCO, CAL.

Dr. Robert Jay, M.D.V., WEST LIBERTY, IA.

### Hoof Diseases.

(Continued from page 550.)

It is base wide, base narrow, toe wide, toe narrow, etc. Study the axis of the foot and hoof—whether it runs normally, or in which direction it deviates. This is important not only in the diagnosis of hoof lameness, but of all other lameness. These points indicate more or less what therapeutic agent is to be employed. This part of the examination for the detection of lameness is, as a rule, utterly neglected, while in reality it is often the key to success.

The horse having thus been carefully sized up, one has now a fair amount of knowledge as to the most probable seat of the lameness, for the reason that certain lameness and certain faulty, or, at least, inferior types of legs and hoofs predispose to certain pathological states.

The examination of the hoof is now in order. Before going into the details of this examination proper, it may be well to allude briefly to the various forms of hoofs, at least the more important ones ordinarily met with.

The large foot is one out of proportion ; it is too voluminous. When met with in the horse worked at a rapid gait the following conditions may be encountered, while the heavy draught is less likely to show them, as it is worked at a slow gait. Concussion being excessive in such a foot, congestion and inflammatory states of the vascular tissues occur, bruises along the bearing surface of the shoe. Animals possessing such a foot are what is vulgarly termed " cows " —that is, they are clumsy, pulling shoes and calking themselves. Usually such a foot is made of brittle horn, the nails holding poorly.

The small foot is one too small for the animal. Such small feet are predisposed to dryness and brittleness. This type of foot stands but little continuous work, breaking down early with footsoreness. (I do not allude to any special disease when using the term foot-sore).

The narrow foot shows an excessive development of the anteroposterior diameter as compared to its transverse diameter. It is predisposed, the same as the small foot, to contraction, quarter-cracks, bar-cracks, navicular disease, ossification of lateral cartilage.

The flat foot is so called on account of the flatness of its plantar surface. As a rule, it is a large foot with oblique walls, low heels, and voluminous frog. When the plantar surface exhibits no concavity at all it is termed a full foot ; while it is known as a pumiced foot when its plantar surface shows a downward bulging that is convexly instead. Such feet, indeed, are predisposed to contusions of the sole, frog, and heels, neat shoeing being a *conditio sine qua non* for their working without lameness. Therefore, loose walls, corns, interfering, calking laminitis, are frequently seen in such feet.

The high-heeled hoof is predisposed to toe-cracks, corns, and bruises in the region of the heels. The low-heeled foot is very sensitive to bruises and contusions of the vascular tissues, quarter-cracks being of frequent occurrence.

These few remarks in regard to the possible diseases of the various hoofs tend to show the importance of being acquainted with the weak points of each hoof. It is, to say the least, disagreeable to be unable to give a definite opinion as to the value of a certain hoof when brought to us for an examination for soundness. I knew of a young veterinarian who condemned a high-priced horse on account of its low-heeled weak feet. The owner of the sale-stable became very abusive and suggested to the intending purchaser to call another veterinarian to settle the question. The writer was selected for this thankless job, and had the pleasure to support the young and by this time much-abused veterinarian in his prognosis, which was " quarter-crack before he is a year older." I took the liberty to add that such forefeet might possibly show seams the first trip the horse would take over the stones. A few days afterward the horse was operated on both fore feet by the writer for quarter-crack. The purchaser at the time of the sale agreed with the seller that no living man could tell when a quarter-crack was due. A law-suit, expert veterinary testimony (oh, ye gods !) wound up this case.

A light-weight hammer and pincers with jaws which open sufficiently wide to easily grasp any two points of the hoof are requisites in the examination of hoof diseases. Supporting leg-lameness being present, and other diseases of the bony column and inhibitory apparatus exhibiting supporting leg-lameness being excluded, the examination of the hoof is indicated.

First, inspect the posture of the patient. Does he toe-out or toe-in or stand straight? Does he point? Has he a large, small, narrow, high-heeled, or low-heeled hoof? Are both feet of the same size? Does he shift the weight often from one foot to the other? Go over every part of the hoof thoroughly and compare it with the same part of the other foot. Having thus thoroughly inspected and compared the hoof as a whole, the quarters, region of the toe, coronet, heels, frog, directions of the bars, condition of the sole, the shoe, the site of the nails, are taken into consideration. The next and most decisive step lies in palpation of the hoof. Here the hand, the hammer, and the pincers are employed. A number of diseased states might have been mentioned under inspection of the hoof, but since palpation would have to be employed to confirm the impressions made upon the visionary apparatus, I shall discuss them under palpation.

The first step is taken to decide whether an inflammatory state of the horn-producing element, the pododerm, exists. For this purpose the sound foot is raised and the pulsations of the metacarpal or digital artery of the lame foot taken. It is absolutely necessary to raise the other foot off the ground when taking the blood-wave of either of the arteries mentioned, as in this way the skin, tendon, etc., of the lame leg are rendered tense, thus giving the arteries a firm foundation, permitting of an exact pulse-taking. There are instances—I refer to the thick-skinned lymphatic animal—where it is impossible to detect any pulsation in either the large metacarpal or digital artery unless the opposite leg is held off the ground by an assistant. Of course, this method of taking the pulse is only necessary in the anterior extremities, not being essential to obtain satisfactory results in the hind legs. On the whole, it is physiological to perceive a limited pulsation along these arteries, this pulse being more marked in the finely-bred, thin-skinned creature than in the tough-hided, lymphatic individual. The beginner will do well to study the pulse in the healthy hoof. Here, again, the writer wishes to draw attention to the fact that in such cases as in all others where room for doubt exists the result obtained by the examination should be compared with the same part of the healthy foot. At the same time increased pulsation does not always mean an inflammation of the pododerm. The increased pulsation is due to an obstruction of the peripheral vessels. The vascular and soft tissues within the unyielding horny capsule, the hoof, are pressed upon and a venous stasis primarily arises. The greater the inflamed area the greater the pulsation of the artery. As stated a moment ago, increased pulsation, while suggestive of a pododermatitis, may follow some other circulatory impediment, thus swelling of the coronary region from a blister or some other disease—disease anywhere along the phalanges as long as accompanied with swelling will of necessity bring with it an increased pulsation of the arteries previously mentioned. Yet practice will enable one to differentiate between the actual throbbing of the artery in a case of true pododermatitis, and the mere fulness of the vessel shows it to depend on a swelling of the subcutis somewhere along the phalanges. To the beginner the following rule is a safe one: "A positive diagnosis of an acute inflammatory process within the horny box can be made if there is an absence of swelling along the phalanges and presence of increased throbbing in the arteries along the fetlock of the lame hoof." It would require more space than a paper of this sort permits to go into the details of the many specific troubles along the phalanges which may lead to diagnostic errors, the writer having dealt with them exhaustively in his work on *Clinical Diagnosis of Lameness in the Horse.* Finally, it is to be remembered that certain hoof diseases, as, for instance, chronic navicular arthritis, do not give rise to increased pulsation of the metacarpal or digital arteries.

The examination having shown excessive pulsation of arteries supplying the hoof with blood, further an absence of swelling along the phalanges, one may be reasonably sure of an acute hoof disease. Now the hoof is placed within both hands, the examining person facing the animal. By placing the hand lightly upon the various parts of the hoof an increase of temperature may be appreciated. The posterior parts are naturally a little warmer than the region about the toe, as they are made up of thinner horn and are more highly endowed with blood supply. While comparing the heat of one part of the hoof with the corresponding part of the other hoof it is well to use one hand only. Should it be desirable to use both hands, then care should be taken that both hands are equally warm, as necessarily very different results will be obtained if one of the examining hands is cold while the other one is warm.

---

Dr. A. G. Kern, of Knoxville, Tenn., takes an active interest in army legislation, and keeps his associates in eastern Tennessee aggressive and earnest in the work of securing favorable consideration at the hands of "Big Bend State's" Congressional representatives.

